# Using Bayesian statistics for longitudinal modelling and individual prediction in neuroimaging

**DOI:** 10.1002/alz.085188

**Published:** 2025-01-09

**Authors:** Roser Sala‐Llonch, Agnès Pérez‐Millan

**Affiliations:** ^1^ Institute of Neurosciences. Department of Biomedicine, Faculty of Medicine, University of Barcelona, Barcelona Spain; ^2^ Biomedical Imaging Group, Biomedical Research Networking Center in Bioengineering, Biomaterials and Nanomedicine (CIBER‐BBN), Barcelona Spain; ^3^ Alzheimer's disease and other cognitive disorders unit, Hospital Clínic, IDIBAPS, Barcelona Spain

## Abstract

Large neuroimaging datasets play a crucial role in longitudinal modelling and prediction of neurodegenerative diseases, as they provide the opportunity to study biomarker trajectories over time. Noteworthy, the availability of these large datasets coexists with a paradigm shift in the theoretical understanding of these diseases: while classical studies aimed at defining disease signatures as group patterns obtained with static cross‐sectional analyses, novel approaches focus on providing individual predictions in the context of phenotypical and temporal heterogeneity. This scenario is often aggravated by the fact that datasets are not homogeneous and suffer from missing points and noisy data.

Given this complexity, Bayesian approaches appear as an elegant solution to model longitudinal data as they rely on the probabilistic distribution of the data rather than referring to a hypothesis derived from the sampling distribution. Here, we provide empirical examples together with a comprehensive state‐of‐the‐art review and future perspectives on the field.

In the first example, we compare Bayesian and frequentist approaches in a Linear Mixed Effects analysis using MRI‐derived hippocampal volumes, from the Alzheimer’s Disease Neuroimaging Initiative (ADNI) database. We simulate different datasets resembling real‐life clinical scenarios. Our results show that in large homogeneous datasets, both approaches perform similarly in differentiating groups, being the frequentist approach simpler and computationally faster. However, the Bayesian approach was the preferred option with limited datasets with less homogeneous groups and large number of missing data [1].

In another example, we discuss the use of Gaussian Processes (GPs), a Bayesian non‐parametric model estimation technique that generates predictions based on the posterior distribution of functions using predefined kernels [2]. We show novel results on the ability of GPs to identify trajectories within the ADNI database.

Finally, we review the use of machine learning approaches to generate individual predictions. We discuss our own results [3], together with other promising tools that use novel approaches derived from Bayesian statistics that go beyond the classical group classifications [4].

**References**:

[1] Pérez‐Millan A, et al., Sci Rep. 2022.

[2] O'Muircheartaigh J, et al., Brain. 2020.

[3] Pérez‐Millan A, et al., Research Square. 2023.

[4] Azevedo T, et al., Commun Med. 2023

